# Annotations

**Published:** 1894-04-21

**Authors:** 


### Annotations.

### A New Dermatological Society.

There has been a Dermatological Society in existence for some time. A new society has now been founded on dermatological lines. The curious may wonder why this should be. There are two objects for which societies may be founded. One is for mutual admiration or mutual protection of interests; another is for the improvement and diffusion of knowledge. The object of the new dermatological is not mutual admiration or the protection of interests, but the improvement and diffusion of knowledge. The founders of the society have put it in the forefront of their programme that the membership shall be open to all registered medical practitioners in the United Kingdom. This is a distinct challenge to all those limited medical societies which, formally or informally, endeavour to confine their membership to a handful of specialists. Let those societies think for a moment what they are doing. They are endeavouring in a quiet but very determined way to profit by one of the least justifiable methods of the pronounced quack. They are trying to keep their knowledge strictly to themselves. This is exactly what the quack does when he practices with his "secret" remedy. Remedies, or methods of treatment which are not communicated freely to the whole profession but kept within a limited circle of a score or a hundred individuals, are for all practical purposes "secret" remedies; and those who bolster up such methods of practice are, we repeat, distinctly treading in the footsteps of the "quack." From all taint of this kind the new Dermatological Society is absolutely free. The first meeting of its members was held at 15, Cavendish Square, on Thursday in last week, and among its leading spirits are Mr. Jonathan Hutchinson, Sir William Broadbent, Dr. Pye Smith, Dr. Liveing, Dr. Radcliffe Crocker, Mr. Malcolm Morris, Dr. McCall Anderson, and the best known British dermatologists of the day. The full title of the new association is the Dermatological Society of Great Britain and Ireland.

### Diphtheria at Leavesden.

In the schools at Leavesden, Herts, where the parish of St. Pancras brings up its poorer children, there has been an outbreak of diphtheria, lasting for several weeks, during which forty-eight children have been attacked by the disease, six have died, three have been removed, and one is now suffering from post-diphtheritic paralysis. Now that the mischief has been done, the cause is being searched for, on the principle, apparently, that it is better to lock the stable door after the horse is stolen than not to lock it at all—a perfectly sound principle when, as in this case, you are dealing with stables of more than one stall. Dr. J. F. J. Sykes, St. Pancras's own medical officer of health, has been sent down to Leavesden, and, in conjunction with Mr. Adams Clarke, the medical officer of the school, has made the usual thorough investigation. The water, the drainage, the milk, and other ordinary founts of origin have all been tapped, but have yielded no definite result. External causes not being discoverable, Dr. Sykes turned his attention to the normal health of the children and the normal sanitation of the schools and their surroundings. Here, it seems to us, he has put his finger upon the right spot. Large tonsils, relaxed throats, weak throats he found everywhere prevalent. In the buildings he discovered localised overcrowding, an unwholesome system of warming the various parts of the school, defective ventilation in parts, and a few closets in immediate contiguity to class-rooms. What need to go further? The children's throats were like fires all laid and waiting the touch of the burning match. Once lighted up, the blaze spread to surrounding susceptible throats, until every feeblest and weakest-throated child in the school was down with the malady. The moral is very obvious. The weakly children should be better fed and raised to a higher tone of health; the schools should be enlarged and overcrowding made impossible; the closets should be strictly cut off from the class-rooms and dormitories, and free and cross ventilation should be provided throughout the buildings. Dr. Sykes has, perhaps, rendered more service to sanitation and the public health than he is aware of, by shewing all and sundry that if they would but take care of the normal, the abnormal would very often take care of itself.

### Huxley on Head and Heart.

Professor Huxley told the members of the Salters' Company, on the occasion of their quincentenary celebration the other day, that he had the greatest possible respect for what was called the "great heart of the people," but he had no great respect for what he might venture to call the "great head of the people." Most readers will regard this as a clever epigram and think no more of it. But those who have been at the trouble to study and understand Professor Huxley's character and words will take a very different view of the matter. The Professor, we know, has never been an ardent admirer of the classes. His opinions of the masses may therefore be regarded as the utterances of a quite dispassionate, scientific observer. In his judgment, after a long life of honest and thorough scientific observation of man, physical, mental, and moral, the great head of the people is neither strong nor wise, though the great heart is exceedingly sound. In other words, the instincts of the ordinary Englishman are honest and honourable, but his knowledge is small, his experience limited, and his judgment, therefore, not always to be relied upon. This, we take it, is a most sober and sensible view of the situation. The Professor draws the natural corollary from it. Seek out your capable men, brother Englishmen, he says in effect. Do not be jealous of them; do not snub them; do not resist them; seek them out; give them every furtherance of education; give them responsibility; give them leadership; pay them generously; let them have free minds to devote to the service of their country. In puissant, honest, unfettered individualism, Professor Huxley sees object lessons, leadership, and progressive improvement for the masses and the whole nation. In the suppression of capable individualism and the artificial equalising of those whom physiology and nature have for ever made unequal, he sees the triumph of stupidity and incapacity, and the imminence of a common ruin. Most undoubtedly he is scientifically and historically right, as right as ever a man was in the world. Patriotism, science, religion, and the enthusiasm of humanity, must all stand shoulder to shoulder for the preservation of a saving individualism.

